# Manual of Diseases of the Skin, from the French of M. M. Cazenave and Schedel, with Notes and Additions

**Published:** 1852-04

**Authors:** 


Art. V.—*Manual of Diseases of the Skin,* from the French of M. M. Cazenave and Schedel, with notes and additions. By Thomas H. Burgess, M. D., Surgeon to the Blenheim Street Dispensary for Diseases of the Skin etc. Second American edition, enlarged and corrected from the last French edition, with additional notes. By H. D. Bulkley, M. D., Physician of the New York Hospital; Fellow of the College of Physicians and Surgeons, New York: Lecturer on Diseases of the Skin, etc., etc. New York: Published by Samuel S. and William Wood: 1852. p. p. 348, 800.

It is a remark of physiologists that as we ascend in the animal scale, we meet, at every step, with a greater specialization of function. In the lowest classes, many functions are performed by a single organ, that which digests the food, for example, being at the same time the organ of respiration, and the oral orifice serving not only for the reception of the food, but also for the expulsion of the ova and of the unassimilable parts of the aliment. In others again, the organs of locomotion are those by which the animal captures and secures its prey, or by which it aerates its blood, by a process analogous to respiration in the higher tribes. The paw of carnivorous quadrupeds, which is their instrument of prehension, is also an organ of locomotion, and in the monkey tribe the anterior extremity still subserves the same end; it is only in man that it is completely liberated and set apart for a single specific purpose.

This same subdivision of labor we witness in the progress of society. During its ruder stages, each individual finds it necessary to perform many offices which, in a more advanced stage, he relinquishes to others. The barber and the surgeon were once united. Until the arts are somewhat advanced, one must be his own cobbler and tailor, and, after a sorry fashion, attempt many things which come to be skilfully executed when labor is wisely divided.

In no calling have the happy results of this principle been more strikingly manifested than in our profession. It is long since the responsible duties of the surgeon were discharged by the barber, but it is not long since dentistry was a regular part of the physician's business; and even now in nearly every portion of our country the doctor is at the same time physician, accoucheur, and surgeon. The tendency everywhere is to subdivision. It has been fully established by experience and observation, that eminence and skill are only to be attained by directing the energies of the mind to a limited field—that

> "One science only will one genius fit,
> So vast is art, so narrow human wit;
> Not only bounded to peculiar arts,
> But oft in these confined to single parts."

The healing art is confessedly quite too vast for any human genius however comprehensive. It is to be cultivated with success by taking some branch of it; its rapid progress in latter times is to be ascribed to the partition of its various subjects among numerous cultivators. First, surgery, midwifery, and medicine have been separated, and each made a special pursuit. Then particular provinces in the several divisions have been made a special study. The eye, the uterus, the brain and the chest have severally occupied some of the best minds of the profession, and who can not see that it is by this devotion to a specialty that our knowledge on all these subjects has been so much increased?

The work before us prefers its claims to public favor upon the ground, that its authors have made the study of cutaneous diseases the business of their lives. It embodies the extensive experience of M. Biett, who, in his day, enjoyed the highest reputation as an authority on diseases of the skin; and to this is added whatever has since been discovered in the practice of others who have devoted themselves to the investigation of this class of affections. The authors, M. M. Cazenave and Schedel, were pupils of Biett, and Cazenave now fills the office occupied for so long a period by his preceptor, at the Hospital of St. Louis, appropriated specially to these diseases. The translator, too, is connected with such a hospital, and Dr. Bulkley has for many years paid particular attention to these disorders. With all these advantages it would have been surprising if the authors had not produced a treatise of uncommon excellence.

The classification they adopt is essentially the one proposed by Willan, which is at the same time simple and scientific. They divide these complaints into the following orders, to wit: *exanthemata*, *vesiculæ*, *bullæ*, *pustulæ*, *papulæ*, *squamæ*, *tubercula*, and *maculæ*; each of which is connected with its own elementary lesion, and presents symptoms of a peculiar type.

Exanthemata.—This term is applied to patches of a reddish color, varying in intensity, size, and form, disappearing under pressure of the finger, and terminating in delitescence, resolution, or desquamation.

Vesiculæ.—A vesicle is a slight elevation of the epidermis, containing a serous and transparent fluid, which, however, is occasionally opaque or sero-purulent. The vesicle may terminate in absorption of the fluid, slight desquamation, excoriation, or the formation of small, thin incrustations.

Bullæ.—Generally speaking, bullæ differ from vesiculæ merely in size; they are small, superficial tumors, caused by effusion of serum underneath the epidermis.

Pustulæ.—This term should be strictly confined to circumscribed collections of pus on the surface of the inflamed mucous layer. The contents of the pustules, in drying, produce scabs, and they may be followed by chronic induration, or by red, inflamed surfaces, or sometimes by slight excoriation.

Papulæ.—These are small elevations, which are solid, resisting, and never contain any trace of fluid; they may, likewise, give rise to ulceration, but generally terminate in resolution or furfuraceous desquamation.

Squamæ.—The term squamæ is applied to the scales of thickened, dry, whitish, friable, and degenerated epidermis, which cover minute papular elevations of the skin; they are easily detached, and may be reproduced for an indefinite length of time by successive desquamations.

Tubercula.—These are small, hard tumors, more or less prominent, circumscribed in form, and persistent; they may becmoe ulcerated at the summit or suppurate partially. In this definition we consider tubercles as elementary lesions, and not those which appear after abcesses.

Maculæ are permanent changes of color in certain points of the skin, or in the whole of the cutaneous envelope, but unattended with any general derangement of the health.

We shall not attempt an analysis of the work, a former edition of which has been for a number of years before the profession, but since the chapter added by Dr. Burgess, on *Glanders and Farcy*, contains matter not so generally known, we suppose some extracts from it will not be unacceptable to our readers.

Essentially these diseases are one. They have long been known in horses, but it is not a great while since they were shown to be communicable to men. It had been frequently remarked that wounds resulting from the posthumous examination of glandered horses, gave rise to serious consequences, but the results were attributed merely to a septic poison analogous to that produced by other putrid matters. In 1812, Lorin published a paper in the *Journal of Veterinary Medicine*, in which he contended that *farcy* might be transmitted from the horse to the human subject, and cited a case in proof of the doctrine. A more detailed case was published by Shilling, a veterinary surgeon at Berlin, in *Rust's Magazine* for 1821. The subject was a stable-boy, at a veterinary college, who became unwell soon after washing the nostrils of a glandered horse. A pustular eruption broke out on the skin, pimples appeared on the nose, which speedily became gangrenous; the boy died, and at the examination of the body after death, small purulent spots were found on the frontal bone, and pus in the muscles of the extremities.

A second case was reported in the same journal, in connection with the foregoing, in which the disease, having been contracted from a glandered horse, terminated fatally on the fourteenth day from the commencement of the attack. Soon after these cases were published in Germany, Mr. Muscroft recorded in the 19th volume of the *Edinburgh Journal*, the case of a jockey who wounded himself in the hand while trimming a glandered horse, and died with all the symptoms of glanders. Other cases were added, from time to time, and the disease was alluded to by Mr. Travers, in 1826, in his work on "*Constitutional Irritation;*" but still the subject had attracted little attention until Dr. Elliotson published his memoir on "*Glanders in the Human Subject,*" in the *Med. Chir. Transactions* for 1830. Up to this time doubts were entertained by many physicians as to the identity of the disease in the two classes of subjects; so that we may say the commencement of our inquiries into the affection, so far as the human being is concerned, dates from the publication of that interesting memoir. In 1833, Dr. Elliotson attended a case with Mr. Youatt, the eminent veterinary surgeon, who, up to that time, would not admit the transmissibility of glanders from the horse to the human subject. We learn from Dr. Burgess, that he was fully convinced by this case, and now believes with all medical men in the transmission of the contagion by this method.

In the *London Medical Gazette* for April, 1840, a case is recorded of a man who died of glanders, and what is remarkable, the nurse who attended him took the disease and also died. This is the only case yet reported, in which the disorder was communicated from one human being to another. The description given by Dr. Burgess of the disease is as follows:

*Symptoms.*—The symptoms of acute glanders in man are essentially typhoid. The disease usually commences with general constitutional disturbance; headache, depression of spirits, prostration of strength, stiffness and constant pain of the joints, aggravated by motion, irritability of the stomach, and excessive thirst. The patient complains of great heat about the nose and windpipe, accompanied with a copious viscid discharge, and with pain in the head, back, and limbs, and constriction about the chest. After a certain period, the nose and surrounding parts become swollen, hot, excoriated, and of a bright red or livid color: one or both eyes are inflamed, or completely closed; a profuse tenacious mucus, at first of a deep yellow, but afterwards of a bloody or dark sanious appearance, exudes from the nostrils, and occasionally from the eyes; hard, round, phlyzacious pustules appear on different parts of the body; the temperature of the skin is increased; the pulse is rapid, soft, and weak or undulating; respiration quick, weak, and shallow. The tongue dry, rough, and reddish brown; the body is bathed in copious and offensive perspiration, the thirst unquenchable, the stools are slimy, and horribly fœtid; the voice is weak, and the mind wandering. In the course of a few days these symptoms become still more aggravated; diffused abscesses appear in various parts of the body, especially about the joints. The fever assumes a more malignant character; the disease extends to the air-passages and lungs; fresh abscesses form and suppurate; the nose and surrounding parts become gangrenous; the perspiration is more profuse and sour; finally, a state of general collapse ensues, and death is ushered in by a low muttering delirium; the fœtor from the discharges, and from the whole body, towards the close of the disease, is insupportable.

When the disease is complicated with farcy, constituting the variety called *farcy glanders*, we may observe the following additional symptoms:—Small tumors on different parts of the body, but more numerous on one side than on the other, having a glossy red appearance, which soon changes to a dark brown. They also affect the head, or even the face, and chiefly on one side; they are sometimes exceedingly painful, they crack on the surface, and a thin acrid sanies exudes. They vary in size, and are generally accompanied with pustules in different parts of the body; the fauces are injected with blood, and of a purplish hue. The inflammation of the lymphatic vessels and glanglions is generally accompanied with diffuse inflammation of the subcutaneous cellular tissue. If the disease be inoculated, as it commonly is, a true pustule sometimes forms in the vicinity of the puncture, to which succeeds an ill-conditioned ulcer, with raised edges and of a greyish aspect. An inflamed red line, or cord, produced by the swollen and inflamed lymphatics, is then observed along the limb, and the lymphatic glands of other parts of the body become sympathetically affected. Simple farcy may thus slowly, but steadily, proceed to the destruction of life, or acute glanders may supervene and hasten that event.

The disease is eminently contagious, and the poison may be communicated through the medium of the atmosphere as well as by contact with a diseased animal.

A gentleman of fortune, in the west of Ireland, had had his stud of horses infected with glanders. Every particle of woodwork in the stables, including stalls, rack, manger, &c., was taken down or replaced with new materials. The plastering on the walls was completely removed, and the pavement ripped up, and all was replaced with entirely new work; but the first horses that were again put into those stables became *infected*, and they were ultimately razed to the ground. It would even appear that the contagious principle remains for a lengthened period, sometimes for years, in any stable or shed where glanders or farcy may happen to have been developed.

M. Hamont has investigated this singular affection with great care, and the conclusion of his researches is, that "the old notion of glanders being always the result of damp, narrow, and ill-ventilated stables, is erroneous."

He maintains, 1. That the original causes of glanders and farcy do not exist in stables. 2. That the habitation exerts but a very secondary influence towards their development. 3. That an insufficiency, or a bad quality of food, may excite both glanders and farcy in degenerated animals; and lastly, that they never appear spontaneously in the blood-horse when well fed and taken care of.

The matter of a glandered sore will produce *farcy*, and that of a farcy-bud will produce *glanders*, a convincing proof of the identity of these diseases.

In regard to the termination of the disorder, Dr. Burgess remarks—

The prognosis of the acute varieties of glanders is highly unfavorable. In the chronic state, life may be prolonged for a certain period, but in such a condition that death would be preferable to it. In the horse, however, this form is not so unfavorable, for the animal may still continue to work, with farcy buds of considerable size along the legs, without the health being seriously injured, and the tumors may ultimately disappear. Although cases of "cure" have been recorded, I doubt very much if they were cases of real glanders, for, as far as our present knowledge goes, glanders still appears to be an incurable disease.

We recommend this treatise to our readers with entire confidence, as one which will prove a useful guide in the diagnosis and treatment of a class of diseases of very frequent occurrence, and often of an exceedingly perplexing character.

